# Case Report: COVID-19 Presenting as Acute Undifferentiated Febrile Illness—A Tropical World Threat

**DOI:** 10.4269/ajtmh.20-0440

**Published:** 2020-05-15

**Authors:** Surat Nunthavichitra, Suttiporn Prapaso, Viravarn Luvira, Sant Muangnoicharoen, Pornsawan Leaungwutiwong, Watcharapong Piyaphanee

**Affiliations:** 1 Department of Clinical Tropical Medicine, Faculty of Tropical Medicine, Mahidol University, Bangkok, Thailand;; 2 Department of Microbiology and Immunology, Faculty of Tropical Medicine, Mahidol University, Bangkok, Thailand

## Abstract

We report a young Thai man from the Thai-Myanmar border suffering from 2 days of fever and myalgia without respiratory tract signs or symptoms. He reported no history of travel through an area with confirmed COVID-19 cases or contact with sick persons. After excluding malaria and dengue, which are common causative agents of acute undifferentiated febrile illness (AUFI) in Thailand, chest radiography was performed according to the patient triage protocol of our institute for AUFI during the COVID-19 outbreak. Chest radiography revealed findings compatible with pneumonia. Nasopharyngeal, throat, and sputum samples tested positive for SARS-CoV-2 by real-time reverse transcriptase–PCR. The preadmission diagnosis of COVID-19 in this patient enabled appropriate management and isolation to prevent nosocomial transmission. Fever and nonspecific symptoms and laboratory results in early COVID-19 may be difficult to distinguish from tropical infectious diseases, especially when respiratory signs and symptoms are absent. This fact necessitates vigilant awareness in clinical investigation, management, and infection control, especially in tropical resource-limited settings.

## INTRODUCTION

Diagnosis of acute undifferentiated febrile illness (AUFI) has been a challenge and burden in clinical practice in the tropics.^1^ Most tropical infectious diseases, including malaria, dengue, leptospirosis, and rickettsioses, present with fever and nonspecific signs and symptoms. Basic laboratory results with these diseases are nonspecific, and accurate point-of-care diagnostic tests may be lacking. The outbreak of COVID-19, due to infection with SARS-COV-2, was identified in December 2019 in Wuhan, China, and has become pandemic.^2^ COVID-19 can present with a wide range of symptoms and severities; fever is the most common symptom and sign.^3^ Thus, COVID-19 cases that present with fever alone may be difficult to distinguish from other AUFIs in the tropics. We report a COVID-19 case presenting as AUFI from the Thai-Myanmar border and discuss this diagnostic challenge.

## CASE REPORT

**Figure 1.**
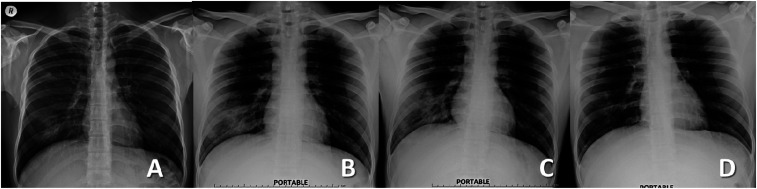
Chest radiography on days 3 (**A**), 5 (**B**), 11 (**C**), and 19 (**D**) of illness.

A 30-year-old Thai man who lived in Tak Province, in northern Thailand, on the Thai-Myanmar border, traveled to a suburb of Bangkok on March 22, 2020. On April 1, he presented with fever and myalgias for 2 days. He denied respiratory tract symptoms or loss of taste or smell. The patient denied visiting an area with COVID-19 cases and did not know of any recent contacts with sick people. He had a body temperature of 38.0°C, blood pressure 108/67 mmHg, respiratory rate 20/minute, pulse rate 99/minute, and room air oxygen saturation 98%. Physical examination revealed no rash or eschars, no lymphadenopathy, normal auscultation of the chest, and no hepatosplenomegaly. Basic laboratory findings were nonspecific: hemoglobin 13.4 g/dL, normal white blood cell and platelet counts, and mildly elevated liver enzymes (aspartate transaminase 44 U/L and alanine transaminase 87 U/L). Screening was performed for common causative agents of AUFI. Point-of-care tests for dengue (NS1 antigen) and malaria (thick smear) were negative. After these negative results, chest radiography (CXR) performed, according to the hospital triage protocol for AUFI to screen for COVID-19, indicated alveolar infiltration of the right lower lung (RLL) (Figure 1A). He was diagnosed with atypical pneumonia, empirically treated with azithromycin, and tested for SARS-COV-2. Real-time reverse transcriptase–PCR (rRT-PCR) tests of nasopharyngeal, throat, and sputum specimens were all positive for SARS-CoV-2 (Detection Kit for Novel Coronavirus 2019-nCoV) RNA; DaAn Gene Co., Ltd., GuangDong, China) and negative for other respiratory pathogens (Allplex™ Respiratory Panel Assays; Seegene Inc., Seoul, South Korea).

Four days after the onset of fever, with the diagnosis of COVID-19 pneumonia, the patient was hospitalized, with close monitoring and isolation. He was treated with azithromycin (500 mg/day), chloroquine (500 mg twice daily), and darunavir plus ritonavir (DRV/r: 600/100 mg twice daily) according to the Thai National Guidelines for COVID-19 diagnosis, treatment, and care.^4^ The fever persisted, with a slight decrease in oxygen saturation (96% on ambient air) on day 6 of illness, and CXR revealed increased patchy ground-glass opacity at the RLL. Treatment with favipiravir (1,600 mg twice daily for 1 day, followed by 600 mg twice daily for 9 days) was initiated on day 8 of illness, and defervescence was achieved the same day. A follow-up CXR was performed on day 11 of illness, which revealed increased patchy opacity at the RLL field, with a cavitary-like lesion in the same area (Figure 1C). Amoxicillin/clavulanic acid was added to treat for possible lung abscess. Despite the progression found on CXR, the patient reported no respiratory symptoms. He had no signs of respiratory distress, and his oxygen saturation on ambient air was 98–99%. Chest radiography on day 14 of illness revealed decreasing patchy opacity in the RLL. All COVID-19 treatments were discontinued on day 19 of illness; on this day repeat rRT-PCR for SARS-CoV-2 of throat and nasopharyngeal swabs were negative, but a sputum specimen was positive. The patient was discharged on day 23 of illness after negative throat, nasopharyngeal, and sputum rRT-PCR tests. On the follow-up at 1 month after the onset of disease, he was without complaints, with almost normal CXR findings.

## DISCUSSION

Acute undifferentiated febrile illness is a common health problem in tropical areas, and a specific differential diagnosis is based on geography. Dengue and murine typhus had to be included in the differential diagnosis of this case because he stayed in a suburban area of Bangkok for a week.^5^ His history of living on the Thai-Myanmar border raised the possibilities of malaria, scrub typhus, and leptospirosis.^6^ Whereas malaria and dengue could be easily evaluated by point-of-care tests, tests for rickettsiosis and leptospirosis were not available. Azithromycin was administered to treat atypical pneumonia, rickettsiosis, and leptospirosis.^7^ The patient came from an area with no known COVID-19 cases and had no known contact with sick persons. Transmission might have occurred during an 8-hour air-conditioned public bus journey to Bangkok.

In a study from China, fever was reported as the most common symptom (88.7%) of COVID-19.^3^ The study reported pneumonia (evidenced by abnormal computed tomography) in most COVID-19 cases, but respiratory symptoms (such as cough) were reported in only two-thirds of cases.^3^ Thus, fever without respiratory symptoms was seen in ∼20% of COVID-19 patients. Fever, rash, and lymphadenopathy, which are common features of AUFI, have also been reported in COVID-19.^3,8^ Overlapping clinical pictures and co-epidemics of COVID-19 with other tropical diseases, such as malaria or dengue, have been a matter for concern in many tropical countries.^9–11^ In addition, the misdiagnosis of COVID-19 due to leukopenia and thrombocytopenia, as well as false-positive dengue IgM, has been reported.^12^ Failure to differentiate COVID-19 from other AUFIs will lead to delays in appropriate management and potential transmission of COVID-19, especially among hospitalized patients.^10^ The exclusion of COVID-19 by rRT-PCR in all fever cases may not be practicable because of limited resources and test sensitivity. According to the national policy at the time of this case, only patients who had fever with respiratory symptoms plus specific risks of potential exposure to COVID-19 required testing to exclude COVID-19.^4^ To raise awareness about COVID-19, the latest national guideline includes screening patients who have fever, respiratory symptoms, and/or exposure risk.^13^

The Hospital for Tropical Diseases in Bangkok established a fever clinic in 2012 to facilitate the management of febrile patients and separate patients with possibly infectious etiologies from other outpatient department care.^5^ Since the start of the COVID-19 outbreak in January 2020, history screening and an isolation room for suspected COVID-19 cases have been implemented. An acute respiratory tract clinic was set up later, according to the national Thai policy. Although a history of exposure could be obtained for most cases in the early stages of the COVID-19 outbreak, it might be missed in some cases, especially with the increasingly wide spread of the disease. Despite the limited sensitivity (69%) of CXR in the diagnosis of COVID-19,^14^ we found it useful for COVID-19 screening.^15^ Thus, our institute has implemented a policy of CXR screening for all AUFI cases, in addition to the national screening guidelines. Furthermore, rRT-PCR for SARS-CoV-2, from swabs and/or sputum, has also been performed in all cases with clinical and/or CXR compatibility with pneumonia for patient triage, to prevent nosocomial transmission of COVID-19. The case reported here supported the implementation of our strong screening policy. Future studies to identify clinical clues to distinguish COVID-19 from other diseases causing AUFI in tropical areas and point-of-care tests for COVID-19 are greatly needed.

In conclusion, COVID-19 needs to be included in the differential diagnosis of patients presenting with fever in tropical areas, even without respiratory symptoms or a history of exposure or travel. Vigilant awareness and investigation are essential for appropriate treatment and infection control for this disease.
